# *Olea europaea* Leaf Phenolics Oleuropein, Hydroxytyrosol, Tyrosol, and Rutin Induce Apoptosis and Additionally Affect Temozolomide against Glioblastoma: In Particular, Oleuropein Inhibits Spheroid Growth by Attenuating Stem-like Cell Phenotype

**DOI:** 10.3390/life13020470

**Published:** 2023-02-08

**Authors:** Melis Ercelik, Cagla Tekin, Gulcin Tezcan, Secil Ak Aksoy, Ahmet Bekar, Hasan Kocaeli, Mevlut Ozgur Taskapilioglu, Pınar Eser, Berrin Tunca

**Affiliations:** 1 Department of Medical Biology, Faculty of Medicine, Bursa Uludag University, 16059 Bursa, Turkey; 2 Department of Fundamental Sciences, Faculty of Dentistry, Bursa Uludag University, 16059 Bursa, Turkey; 3 Inegol Vocation School, Bursa Uludag University, 16059 Bursa, Turkey; 4 Experimental Animal Breeding and Research Unit, Faculty of Medicine, Bursa Uludag University, 16059 Bursa, Turkey; 5 Department of Neurosurgery, Faculty of Medicine, Bursa Uludag University, 16059 Bursa, Turkey

**Keywords:** *Olea europaea* leaf extract, oleuropein, hydroxytyrosol, tyrosol, rutin, temozolomide, glioblastoma

## Abstract

The effects of *Olea europaea* leaf extract (OLE) phenolics, including oleuropein (OL), hydroxytyrosol (HT), tyrosol (TYR), and rutin against glioblastoma (GB), independently and in combination with temozolomide (TMZ), were investigated in T98G and A172 cells. Cell growth was assessed by WST-1, real-time cell analysis, colony formation, and cell cycle distribution assays. A dual acridine orange propidium iodide (AO/PI) staining and annexin V assay determined cell viability. A sphere-forming assay, an intracellular oxidative stress assay, and the RNA expression of CD133 and OCT4 investigated the GB stem-like cell (GSC) phenotype. A scratch wound-healing assay evaluated migration capacity. OL was as effective as OLE in terms of apoptosis promotion (*p* < 0.001) and GSC inhibition (*p* < 0.001). HT inhibited cell viability, GSC phenotype, and migration rate (*p* < 0.001), but its anti-GB effect was less than the total effect of OLE alone. Rutin decreased reactive oxygen species production and inhibited colony formation and cell migration (*p* < 0.001). TYR demonstrated the least effect. The additive effects of OL, HT, TYR and rutin with TMZ were significant (*p* < 0.001). Our data suggest that OL may represent a novel therapeutic approach against GB cells, while HT and rutin show promise in increasing the efficacy of TMZ therapy.

## 1. Introduction

Glioblastoma (GB) is denoted as a highly aggressive primary brain malignancy attributed to its propensity to grow invasively and develop resistance to treatment [1]. Current GB treatment consists of maximum surgical resection, radiotherapy, and chemotherapy [2]. In part because of its invasive nature, failures in surgical resection and subsequent recurrence are documented, such that the mean survival of 14–15 months and a 5-year survival rate of 9.8% is inadequate [3]. Temozolomide (TMZ), an alkylating agent, is the most accepted standard chemotherapeutic agent [4]. However, its effectivity in these tumors is limited due to epigenetic resistance. Further, GB patients often experience various side effects related to TMZ treatment, including nausea, vomiting, fatigue, and hematologic toxicity [5]. In light of the aforementioned inadequacies in the current standard of care for GB, a novel approach to increase the therapeutic effect of TMZ while reducing cellular toxicity is required.

The olive tree *(Olea europaea)* is one of the most-grown trees in the Mediterranean, covering approximately 98% of the world’s crop [6]. The medicinal properties of its leaves are known, with many studies demonstrating not only its nutritional properties but also its curative abilities [7,8]. Olive leaf extract (OLE) contains phenolic compounds widely used in modern pharmaceutical industries, including secoiridoids, flavonoids, and simple phenols with antioxidant, antimicrobial, and antiproliferative properties [9,10]. The primary component of OLE is the secoiridoid oleuropein (OL), comprising approximately 17% to 23% [11,12]. Hydroxytyrosol (HT) is the second major OLE component formed from OL hydrolysis [13]. Both of these are known to demonstrate antioxidant and anti-inflammatory properties [11,12,13]. OLE was also shown to include the antioxidant phenolic rutin [12,13,14] and trace amounts of tyrosol (TYR) with a relatively lower antioxidant effect [6,9]. These plant extract-derived flavonoids have garnered attention by augmenting chemotherapeutic agents’ effects and attenuating their cytotoxic side effects [15]. Studies in the last decade provided evidence of primary GB tumor cell and GB cell line suppression with OLE in adjunct with TMZ [16]. However, further investigation by a study in 2019 highlighted variations in the degree of GB cell affectation of OL alone versus OLE combined with TMZ treatment [17]. As such, further investigation is warranted to clarify the individual effects of the flavonoids within OLE, which contributes to further safe and effective therapeutic approaches against GB.

This study aimed to investigate and compare the apoptosis-promoting and cancer cell growth inhibitory effects of OLE flavonoids, particularly OL, HT, TYR, and rutin, individually and in combination with TMZ against GB cells. The findings of this study are expected to highlight the inclusion of OLE-derived flavonoids as the most suitable precursor candidate in further drug development studies against GB tumors.

## 2. Materials and Methods

### 2.1. Cell Lines and Reagents

Human GB cell lines, the polymorphic with fibroblast-like and spherical T98G and the monomorphic fibroblast-like A172, were gifted by Dr. Tugba Bagci Onder, Koc University, Istanbul, Turkey. A murine healthy fibroblast cell line, L929, was obtained from the American Type Culture Collection (ATCC; Rockville, MD, USA). These cells were maintained using Dulbecco’s modified Eagle medium-F12 (DMEM-F12; HyClone, UT, USA) supplemented with L-glutamine with 10% fetal bovine serum (FBS; BIOCHROME, Berlin, Germany), 1 mM sodium pyruvate, 100 µg/mL streptomycin, and 100 U/mL penicillin. All cells were incubated at 37 °C and 5% CO_2_.

TMZ (cat no: T2577), OL (cat no: 12247), HT (cat no: PHL80152), TYR (cat no: 188255), and rutin (cat no: PHL89270) were purchased from Sigma (St. Louis, MO, USA). OLE was confirmed by botanist Prof. Dr. Gülendam Tumen and sourced from Kale Naturel (lodged: 5 June 2007, voucher specimen number: 10–00,014–00,015–0; Edremit-Balikesir, Turkey). TMZ, OL, HT, and TYR were dissolved in dimethyl sulfoxide (DMSO), while rutin was dissolved in water according to the manufacturer’s instructions. OLE was freshly dissolved in water, as described previously [16].

### 2.2. Cell Proliferation and Viability

The cell proliferation reagent WST-1 (Roche Applied Sciences, Mannheim, Germany) was used to determine the effect of flavonoids on the proliferation rate of T98G, A172, and L929 cells. Briefly, 2 × 10^4^ of T98G and A172 cells were pretreated with varying concentrations of TMZ, OLE, OL, HT, TYR, and rutin for 24 h. In contrast, the exact concentration of DMSO used to dissolve phenols that did not exceed a concentration of 0.1% and did not affect cell growth was administered to the untreated cells. After phenol treatments, cells were exposed to 10 µL of premixed WST-1 cell proliferation reagent for 2 h at 37 °C. Quantitative evaluation of cell proliferation and inhibition rates were determined by a microplate reader (Tecan, Switzerland) at 450 nm absorbance., as described previously [18]. Afterward, the toxic effect of each flavonoid was evaluated by applying their 50% inhibitory concentration (IC50) to the L929 cells.

### 2.3. Real-Time Cell Proliferation Monitoring

T98G and A172 cells (15 × 10^3^) were plated onto an E-Plate 16 (ACEA Biosciences, San Diego, CA, USA) with the standard medium. Cells were treated with IC50 doses determined by WST-1 analysis of TMZ-only, OLE-only, and OLE’s phenolic compounds. Cell proliferation indexes were determined every 30 min over 96 h by measuring electrical impedance using the xCELLigence biosensor cell analysis system (ACEA Biosciences, USA).

### 2.4. Colony-Forming Cell Assay

T98G and A172 cells (1 × 10^5^) were treated with OLE-only, TMZ-only, and the different OLE-derived phenolic compounds with and without TMZ. After 24 h of incubation at 37 °C, the cells were harvested, and 1 × 10^3^ of those pretreated cells were cultivated in an individual plate for 10 days. The cell medium was refreshed every 5 days. The colonies formed at the end of the incubation were stained using the CellMAX Colonogenic Assay Kit (BioPioneer, San Diego, CA, USA). These were counted and measured with the following values: size: 0.01-Infinity, circularity: 0.3–1000, using the ImageJ v1.53s software (National Institutes of Health, Bethesda, MD, USA).

### 2.5. Cell Cycle Distribution Analysis

Analysis of the cell cycle was performed using a Muse Cell Cycle Kit (MilliporeSigma, St. Louis, MO, USA) following the manufacturer’s recommendations. A total of 15 × 10^4^ phenol pretreated cells were situated in ice-cold 70% ethanol and were incubated at negative 20 °C for 3 h. These were then stained with 200 µL of the Muse Cell Cycle reagent for 30 min in the dark at room temperature. Cell cycle distribution was analyzed using the Muse Cell Analyzer (Merck, Darmstadt, Germany).

### 2.6. Dual Acridin Orange Propidium Iodide (AO/PI) Fluorescent Staining

A total of 5 × 10^4^ GB cells were plated onto a 24-well plate and treated with IC50 doses of TMZ, OLE, OLE phenolics, TMZ + OLE, and TMZ + OLE phenolics for 24 h. The GB cells were exposed to a dual fluorescent staining solution (100 μL) containing 10 μg/mL AO and 5 μL PI (Sigma, St. Louis, MO, USA) for 5 min. The morphology of apoptotic cells was examined using a fluorescent microscope (EVOS M5000, Thermo Fisher Scientific, Waltham, MA, USA).

### 2.7. Analysis of Apoptosis

Cell viability was analyzed using the Muse Annexin V & Dead Cell Assay Kit (Millipore; Burlington, MA, USA), following the manufacturer’s instructions. A total of 15 × 10^4^ cells were treated with the IC50 doses of TMZ, OLE, OL, HT, TYR, and rutin individually and in combination with TMZ. After 24 h, the cells were harvested and treated with an Annexin V and Dead Cell reagent in the dark for 20 min. Flow cytometry analysis was carried out by a Muse Cell Analyzer (Millipore; Burlington, MA, USA). Cells positive for Annexin V alone were identified as early-apoptotic, whereas cells positive for Annexin V and PI were counted as late-apoptotic. Cells positive for PI alone showed necrosis or nonapoptotic cell death [19]. All experiments were conducted in three technical repeats.

### 2.8. Tumorsphere-Forming Assay

T98G and A172 cells (80 cells/well) were seeded onto 96-well polystyrene microplates coated with ultra-low attachment (PerkinElmer Inc., Waltham, MA, USA), and were maintained in the culture medium (5% CO_2_, 37 °C). After 3 days, the cells formed spheroids, the microscopic images of which were captured. Subsequently, the medium was replaced, and the spheroids were exposed to OLE-only, OLE’s phenolic compounds, or their TMZ combinations for 96 h. Sphere size was measured using the ImageJ v1.53s software (National Institutes of Health, Bethesda, MD, USA).

Live-cell toxicity was analyzed via staining with a mixture of 2 µM of Calcein AM (Abcam, Cambridge, MA, USA) and 4.5 µM of PI (Thermo Fisher Scientific, Waltham, MA, USA). The solution was freshly prepared in sterile phosphate-buffered saline (PBS; Thermo Fisher Scientific, CA, USA). Spheroids were visualized by fluorescent microscopy using an EVOS M5000 Imaging System (Thermo Fisher Scientific, CA, USA). Calcein AM was detected at an excitation/emission wavelength of 488/520 nm, while PI was detected at 535/615 nm.

### 2.9. Real-Time Quantitative PCR

Total RNA was extracted using a Zymo RNA Isolation Kit (Zymo Research, Irvine, CA, USA). RNA quality and concentration were determined by the 260:280 ratio measured by a UV/Vis spectrophotometer (Beckman Coulter, Brea, Canada). A High-capacity cDNA Synthesis Kit (Thermo Fisher Scientific, USA) converted the total RNA (100 ng) to cDNA. The expression of stem-like cancer cell marker genes Prominin-1 (CD133; Hs01009259_m1) and Octamer-binding transcription factor 4 (OCT4; Hs04260367_gH) was analyzed using qPCR. The RNA input was normalized using a housekeeping gene, Glyceraldehyde 3-phosphate dehydrogenase (GAPDH;(Hs02786624_g1). The threshold cycle (Ct) for RNA expression was determined using the StepOne RT-qPCR Real-Time PCR Detection System (Thermo Fisher, CA, USA). Fold changes of Ct values were calculated using the 2^−ΔΔCt^ method [20].

### 2.10. Detection of Intracellular Oxidative Stress Level

The Muse Oxidative Stress Kit (Merck, Darmstadt, Germany) measured reactive oxygen species (ROS), such as superoxide radicals, singlet oxygen, and peroxide, in cells undergoing oxidative stress. A total of 15 × 10^4^/10 µL of cells were harvested in 190 µL Muse Oxidative Stress Reagent (1:8000) and incubated at 37 °C for 30 min. The Muse Cell Analyzer (Merck, Darmstadt, Germany) plotted the histogram of ROS-producing and nonproducing cell populations.

### 2.11. Scratch Wound-Healing Assay

A confluent monolayer of T98G and A172 cells in a 6-well plate was scratched by dragging a 100 μL pipette tip across the thin membrane and was washed twice in PBS to remove cell debris. The wounded monolayers were cultured for 24 h in the presence of OLE-only, OLE’s phenolic compounds, or their TMZ combinations. Microscopic images of the entire wounded area were captured immediately after wounding and again at the end of the 24th hour. Changes in wound size were measured using the ImageJ v1.53s software (National Institutes of Health, Bethesda, MD, USA). Each experiment was performed in technical triplicates.

### 2.12. Experimental Design

**Figure 1:**
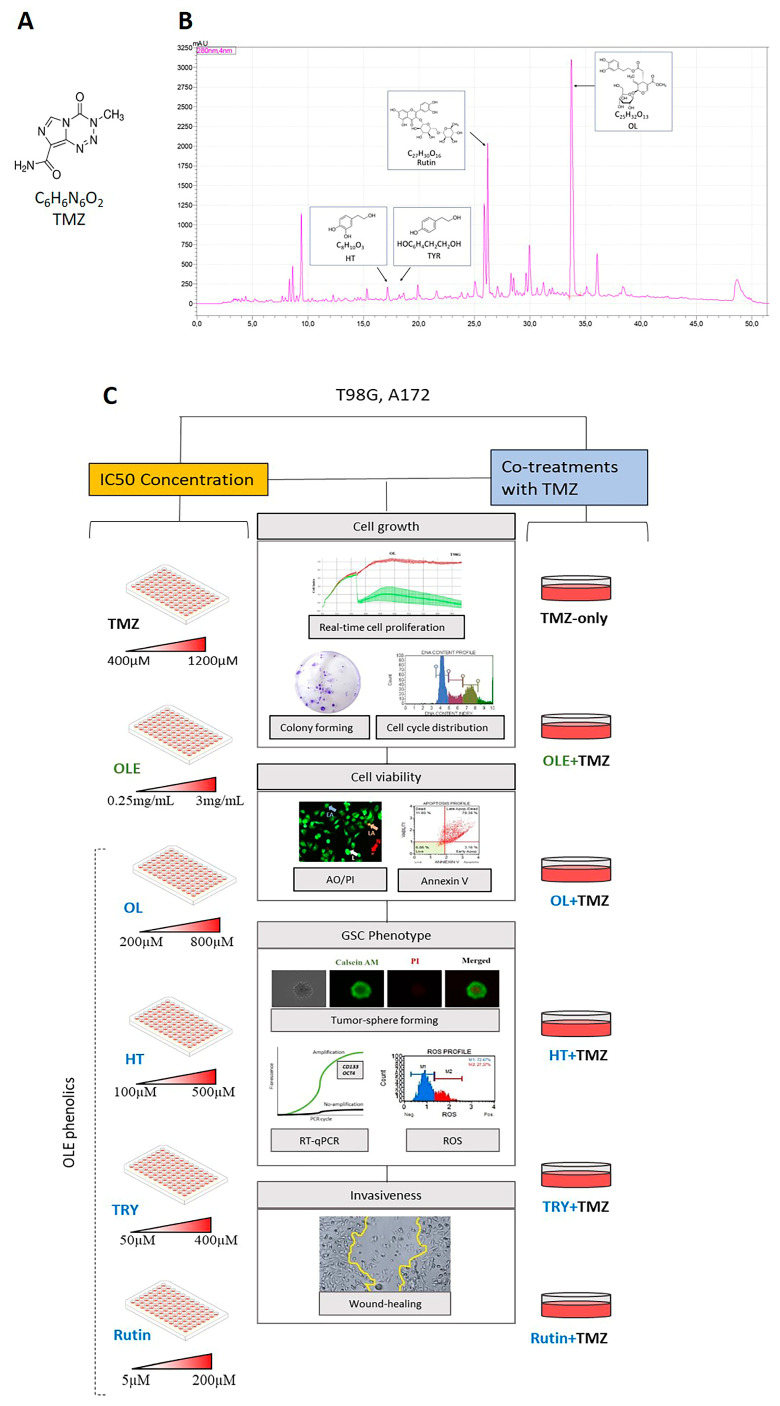
(**A**) ChemDraw structure of TMZ. (**B**) The spectroscopic properties of OLE includes the investigated phenolics, OL, HT, TYR, and rutin. The figure was modified from our previous article from Ercelik and colleagues [22]. (**C**) Schematic presentation of the experimental workflow. A172 and T98G cells were treated with the IC50 concentrations of TMZ, OLE, OL, HT, TYR, and rutin. In addititon, the anti-GB effect of combined TMZ + OLE or TMZ + OL, TMZ + HT, TMZ + TYR, and TMZ + rutin were compared with TMZ-only. TMZ: temozolomide, OLE: *Olea europaea* leaf extract, OL: oleuropein, HT: hydroxytyrosol, TYR: tyrosol.

IC50 concentrations of TMZ, the most common antineoplastic GB medicine [4], OLE, and the phenolics of OLE, including OL, HT, TYR, and rutin [9,10,11,12,13,14], were used against the A172 and T98G GB cell lines [21]. The chemical formulas of the TMZ and phenolic compounds in OLE are shown in Figure 1A,B. An Agilent 1200 HPLC system (Waldbronn, Germany) identified 19.419 mg/mL of OL and 409 mg/mL of rutin in the phenolic compounds of the OLE fractions at 280 nm of wavelength (Figure 1B) [22]. HT and TYR were detected in trace amounts (Figure 1B) [23]. In addition, cotreatments of TMZ + OLE or TMZ + OL, TMZ + HT, TMZ + TYR, and TMZ + rutin were used to observe the combined effect of OLE and OLE phenolics with TMZ (Figure 1C).

### 2.13. Statistical Analysis

Statistical analysis was performed using one-way and two-way ANOVA tests and independent samples T-tests using IBM SPSS Statistics for Windows, Version 20.0 (IBM Corp., Armonk, NY, USA) and GraphPad Prism 8.0 (GraphPad Software Inc., San Diego, CA, USA). Statistical significance was set at a *p*-value < 0.05. All experiments were performed in triplicate, and the data were presented as mean ± SE.

## 3. Results

### 3.1. Active Phenolic Compounds in OLE Inhibit GB Cell Proliferation

**Figure 2:**
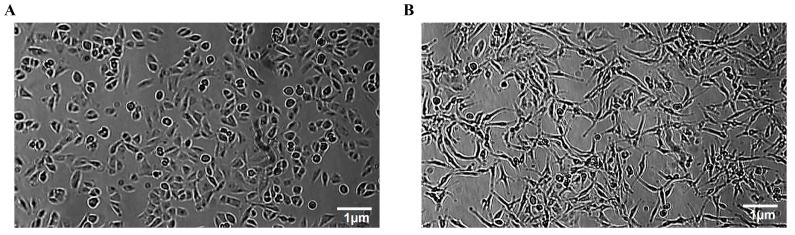
Morphological features of human GB cells. (**A**) Polymorphic morphology of T98G cells consisting of fibroblast-like, polygonal, and spherical cells. (**B**) Monomeric morphology of A172 cells consisting of fibroblast-like cells.

TMZ-sensitive A172 and TMZ-resistant T98G cells were confirmed to be morphologically distinct by microscopy (Figure 2). While A172 cells consisted of monomorphic fibroblast-like cells, T98G cells were seen to be polymorphic with polygonal and spherical cells.

GB cell lines A172 and T98G were treated with a range of concentrations consisting of TMZ-only (400 µM to 1200 µM), OLE-only (0.25 mg/mL and 3 mg/mL), OLE phenolics, including OL (100 µM to 800 µM), HT (100 µM to 500 µM), TYR (50 µM to 400 µM), and rutin (5 µM to 200 µM) for 24 h [16,17,24,25,26].

**Figure 3:**
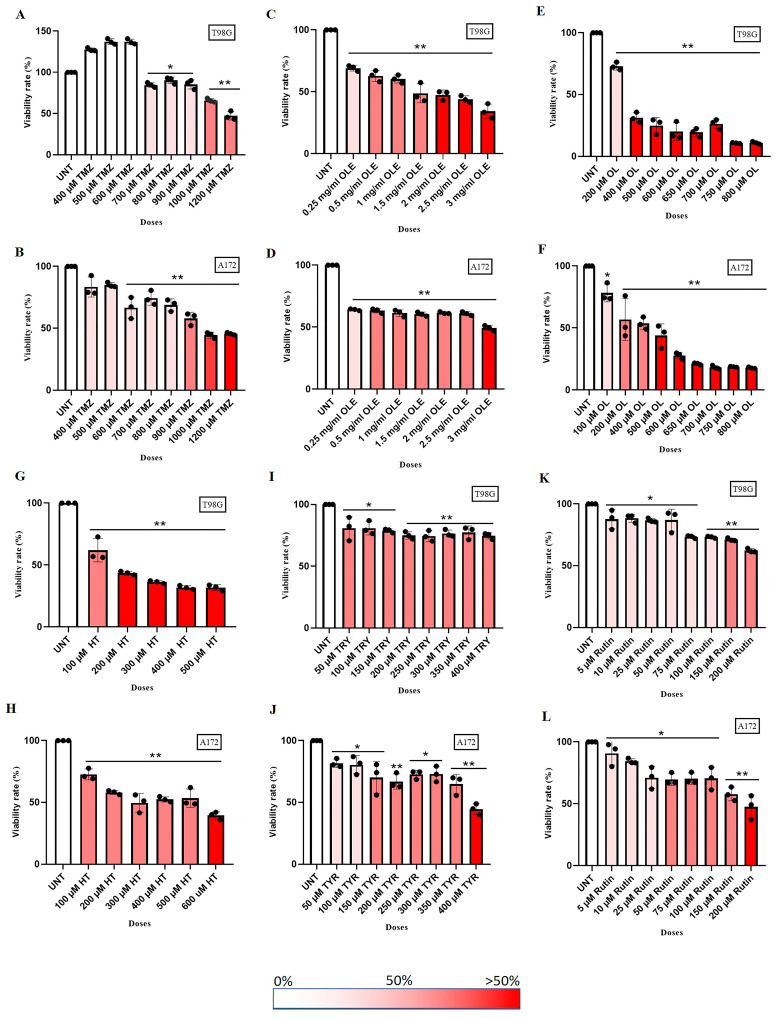
TMZ-only, OLE-only, and OLE phenolics dose-dependently decreased GB cell proliferation. A WST-1 test detected the IC50 dose of TMZ-only (**A**,**B**), OLE-only (**C**,**D**), OL-only (**E**,**F**), HT-only (**G**,**H**), TYR-only (**I**,**J**), and rutin-only (**K**,**L**). A total of 2 × 10^4^ T98G and A172 cells were positioned to 96-well transparent plates and exposed to the tested agents dose-dependently for 24 h. WST-1 activity was recorded at 450 nm absorbance after 2 h of exposure. *p*-values were calculated using one-way ANOVA and Tukey’s post hoc tests (*n* = 3). Data are presented as mean ± SD. * *p* < 0.05; ** *p* < 0.0001 versus untreated cells. UNT: untreated, TMZ: temozolomide, OLE: *Olea europaea* leaf extract, OL: oleuropein, HT: hydroxytyrosol, TYR: tyrosol.

A total of 1000 µM TMZ decreased cell proliferation by 65.9% in T98G and 44.41% in A172 cells (Figure 3A,B). A total of 1 mg/mL OLE was defined as 60.30% and 61.36% inhibitory ability in T98G and A172 cells, respectively (Figure 3C,D). The cell proliferation inhibitory concentration of phenolic compounds of OLE was as follows: 200 µM OL (in T98G: 72.9%; in A172: 56.66%; Figure 3E,F), 100 µM HT (in T98G: 61,94%; in A172: 72,64%, Figure 3G,H), 350 µM TYR (in T98G: 77.35%; in A172: 69.79%, Figure 3I,J), and 150 µM Rutin (in T98G: 71.13%; in A172: 57.81%, Figure 3K,L)

**Figure 4:**
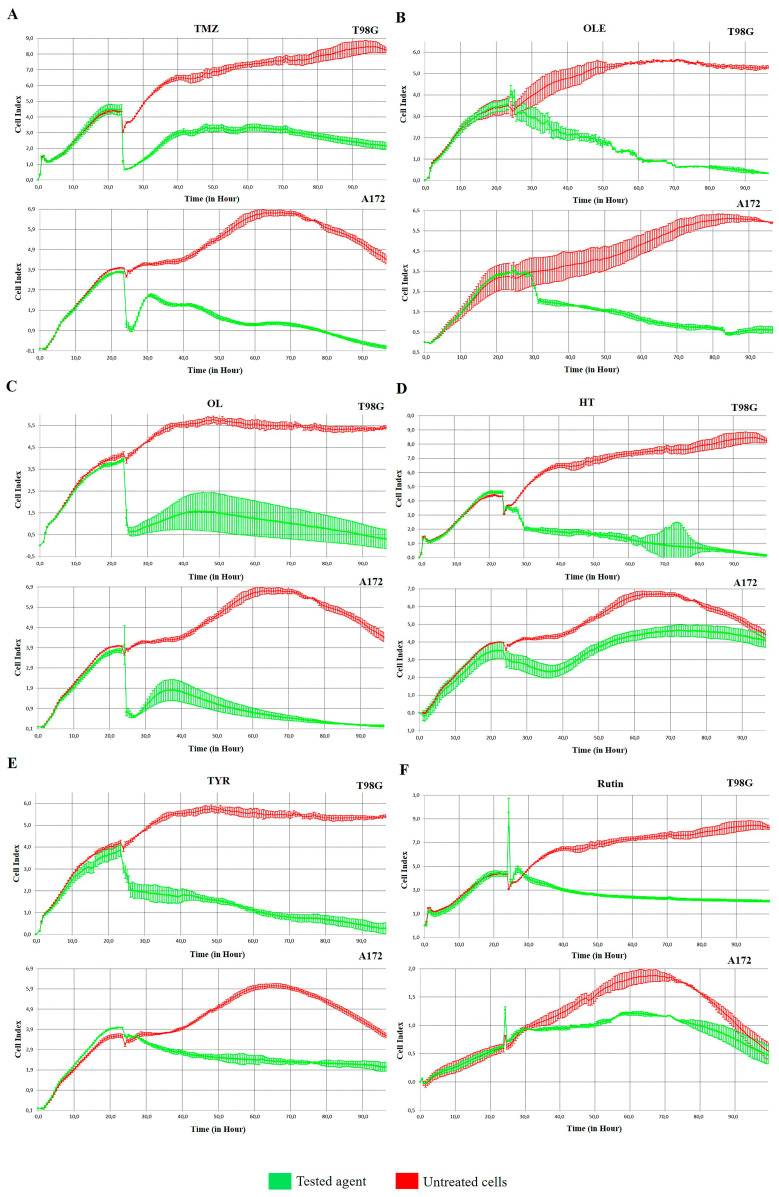
The effect of IC50 doses of TMZ-only, OLE-only, and OLE phenolics on the cell proliferation index of GB cells. (**A**) The effect of TMZ-only (**B**) OLE-only (**C**), OL-only (**D**), HT-only (**E**), TYR-only, and (**F**) rutin-only on T98G and A172 cells. UNT: untreated, TMZ: temozolomide, OLE: *Olea europaea* leaf extract, OL: oleuropein, HT: hydroxytyrosol, TYR: tyrosol.

The IC50 concentration of TMZ-only was detected as 1000 µM for T98G and 900 µM for A172 cells according to their cell proliferation inhibitory capacity. In addition, 1 mg/mL and 0.5 mg/mL were estimated as the IC50 concentrations of OLE-only in T98G and A172 cells, respectively. Likewise, the IC50 concentrations of OLE phenolics for T98G and A172 cells, respectively, were computed as follows: OL-only: 267 µM and 200 µM; HT-only: 91.4 µM and 112 µM; TYR-only: 350 µM and 150 µM; and rutin-only: 142.2 µM and 40 µM. The effect of estimated IC50 concentrations of TMZ-only, OLE-only, and the OLE phenolics on T98G and A172 cells was confirmed by real-time monitoring of cell growth kinetics for 24 h (Figure 4A–F). These concentrations of TMZ, OLE, and phenolic components of OLE were used in all subsequent experiments. It was also noted that for T98G cells, IC50 concentrations of TMZ, OLE, and OLE phenolics interrupted the proliferation of the nontumor fibroblast cell line L929 by less than 50%. Therefore, none of the IC50 doses of the phenolic compounds were cytotoxic in these cells (Figure S1).

### 3.2. Phenolic Compounds Decreased Colony Formation and Reduced G2/M Cell Cycle Arrests

**Figure 5:**
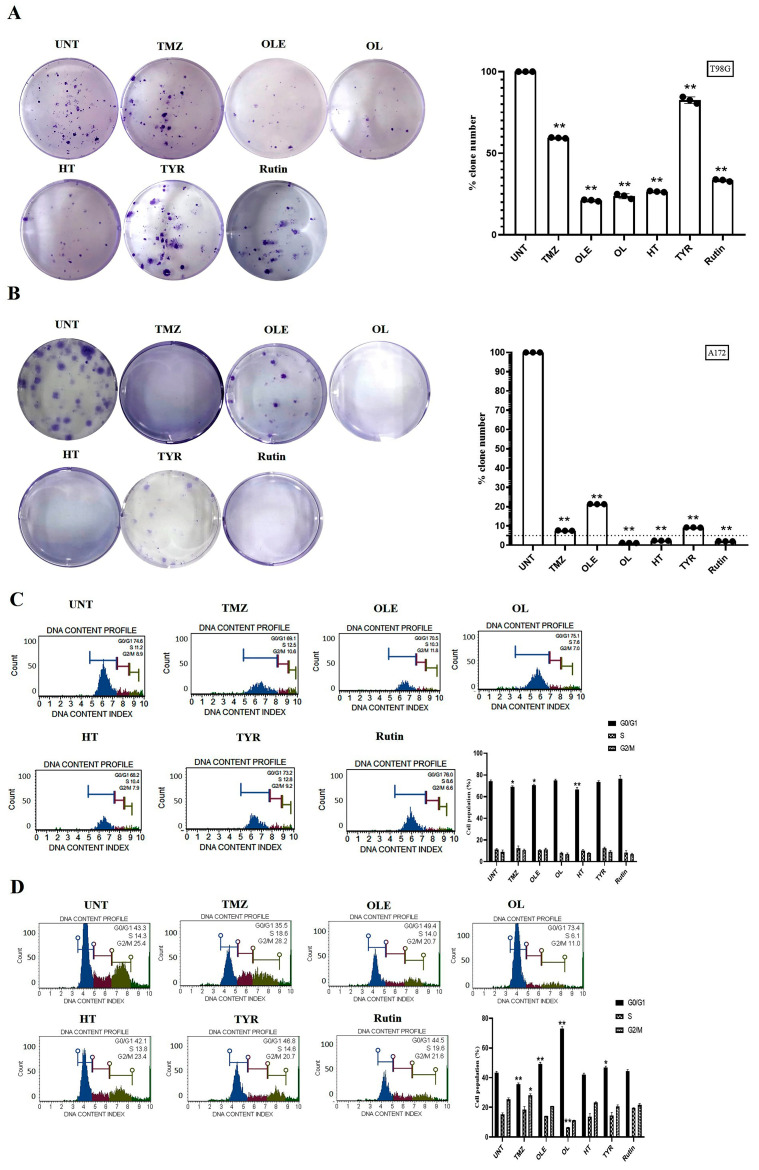
TMZ, OLE, and OLE phenolics decreased the colony-forming ability of GB cells and arrested the cell cycle in the G2/M phase. Colony-formation assays were performed with T98G and A172 cells after treatment with TMZ, OLE, and phenolic compounds. The colony-forming ability of T98G cells (**A**) and A172 cells (**B**) following treatment with 1000 and 900 µM TMZ-only, 1 and 0.5 mg/mL OLE-only, 267 and 200 µM OL-only, 91.4 and 112 µM HT-only, 350 and 150 µM TYR-only, and 142.2 and 40 µM rutin-only. The percentages of the formed colony numbers are graphed. The histograms are of the percentage, mean fluorescence intensity, and %CV of each cell cycle phase (G0/G1, S, and G2/M) in T98G (**C**) and A172 (**D**) cells (n = 3). Data are presented as the mean ± SD. *p*-value was calculated as compared to untreated cells using the two-way ANOVA test (n = 3) * *p* < 0.05, ** *p* < 0.0001 versus untreated cells. UNT: untreated, TMZ: temozolomide, OLE: *Olea europaea* leaf extract, OL: oleuropein, HT: hydroxytyrosol, TYR: tyrosol.

The number of colonies formed by untreated GB cells was normalized to 100%. Compared with normalized colony numbers obtained from untreated cells, TMZ reduced the number of colonies formed by T98G and A172 cells by 1.7- and 13.4-fold, respectively (Figure 5A,B). In addition, after OLE-only treatment, the number of colonies formed by T98G and A172 cells was reduced by 4.6- and 4.7-fold, respectively. The OLE phenolics, OL (4.6-fold), HT (3.7-fold), TYR (1.2-fold), and rutin (2.9-fold) likewise decreased the number of colonies formed by T98G cells (Figure 5A). In A172 cells, colony formation was decreased by more than 40-fold after OL, HT, and rutin treatments, but less reduction (10.84-fold) was observed after TYR treatment (Figure 5B).

Relative to the untreated cells, TMZ did not affect the distribution of T98G and A172 cells within the cell cycle, while OLE decreased the number of T98G cells in the G0/G1 phase (*p* < 0.05) and increased the number of A172 cells in the G0/G1 phase (*p* < 0.0001) (Figure 5C,D). In addition, the OL-mediated induction of the G0/G1 phase was the highest in A172 cells, much more potent than OLE alone. HT and rutin altered the cell cycle by decreasing the number of cells in the G2/M phases in both T98G and A172 cells. However, the effect of HT was less potent than OLE alone in the A172 cell line. In addition, rutin demonstrated a similar cell cycle pattern in T98G cells compared to OLE but was less potent in A172 cells. TYR induced the rate of A172 cells in G0/G1 phases (*p* < 0.05) but did not affect the cell cycle distribution in T98G cells (Figure 5C,D).

### 3.3. OLE Compounds Vary in Decreasing Cell Viability

**Figure 6:**
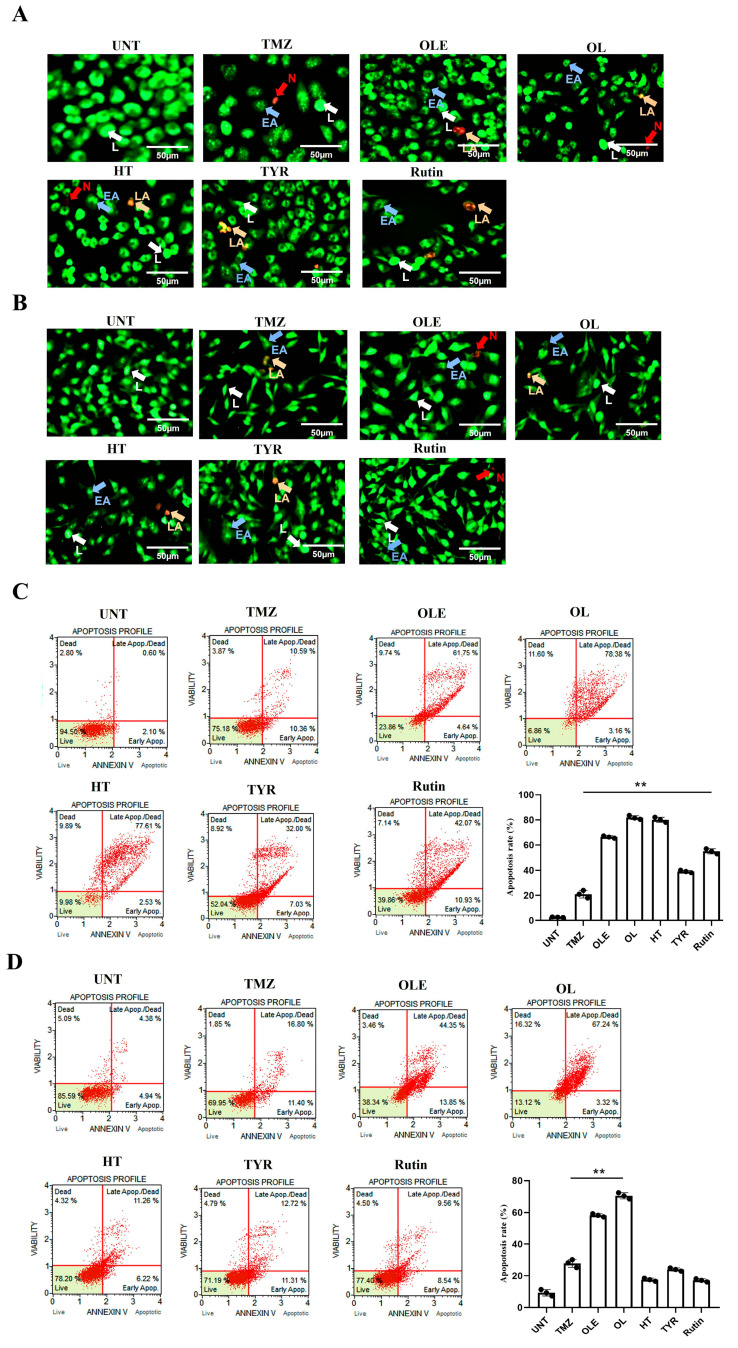
Viability of human GB cells after treatment with TMZ-only, OLE-only, and OLE phenolics. AO/PI staining of T98G (**A**) and A172 cells (**B**). The green cells with a granular nucleus located on one side indicated apoptosis. In contrast, a circular nucleus uniformly distributed in the center of the cell indicated a cell in interphase. The red cells with an inapparent outline indicated necrosis, dissolved or near disintegration. The color-coded arrows indicate the following: alive cells in white, early apoptosis in blue, late apoptosis in orange, and necrosis in red. (**C**) TMZ-only, OLE-only, and OLE phenolics induced apoptosis in T98G and (**D**) A172 cells. The adjusted *p*-values were calculated using one-way ANOVA and Tukey’s post hoc tests. ** *p* < 0.0001 compared to TMZ-only treatment; n = 3. UNT: untreated, TMZ: temozolomide, OLE: *Olea europaea* leaf extract, OL: oleuropein, HT: hydroxytyrosol, TYR: tyrosol. L: alive cells, EA: early apoptosis, LA: late apoptosis, N: necrosis.

Morphological analysis of T98G and A172 cells treated with TMZ-only, OLE-only, and OLE phenolics was performed using the fluorescent dye staining method, Acridine Orange (AO)/PI. The untreated T98G and A172 cells had a uniform circular nucleus in the center. In contrast, the T98G and A172 cell nuclei following treatment with the IC50 concentrations of TMZ-only, OLE-only, and OLE phenolics demonstrated a horseshoe-shaped configuration, a sign of nuclear fragmentation seen during apoptosis (Figure 6A,B). Indeed, according to the annexin V analysis, OLE-only increased apoptosis in T98G and A172 cells (*p* < 0.0001, Figure 6C,D) compared to untreated cells. The apoptosis-inducing effect of OL was higher than TMZ-only in both T98G and A172 cells (*p* < 0.0001) (Figure 6C,D). HT-only treatment resulted in a higher rate of apoptosis than TMZ-only in T98G cells (*p* < 0.0001, Figure 6C) but a lower rate of apoptosis in A172 cells compared to those of TMZ-only treated cells (Figure 6D). Additionally, while TYR and rsutin induced apoptosis in both cell lines compared to untreated cells, these were less effective than TMZ alone in the A172 cell line (Figure 6C,D).

### 3.4. Phenolic Compounds Inhibited 3D Spheroid Growth

**Figure 7:**
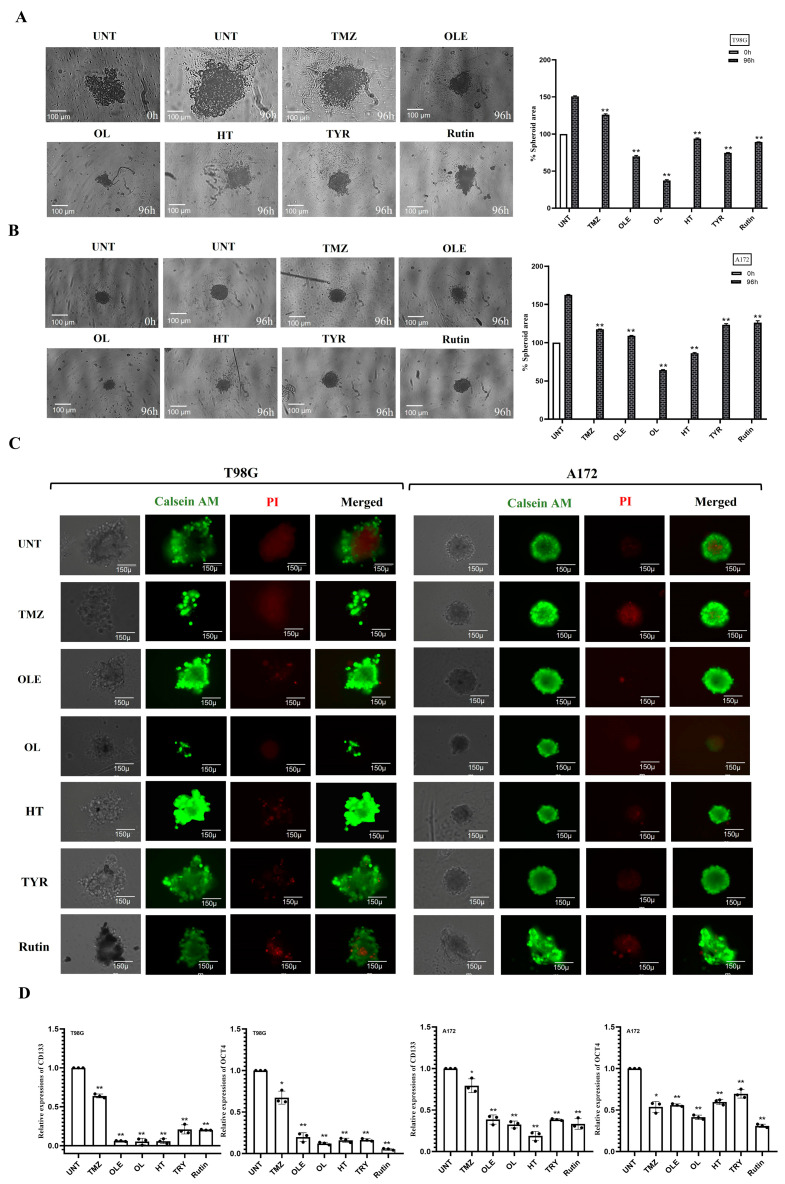
GSC inhibitory capacity of OLE phenolics. (**A**) The effect of TMZ-only, OLE-only, and OLE phenolics on the size of tumorspheres formed by T98G cells and (**B**) A172 cells. Tumorspheres were formed in a 96-well round-bottom ultra-low attachment plate in 3 days. (**C**) Calcein-AM/PI staining determined hypoxia in tumorspheres formed by T98G and A172 cells. Green fluorescence shows metabolically viable and proliferating cells, while red reflects the hypoxia-induced loss of cell membrane integrity and necrotic cells. The tumorspheres were initially imaged untreated and 96 h after treatments with TMZ-only, OLE-only, and OLE phenolics at 40× magnification. ImageJ software measured the size of the captured tumorspheres and the viable/necrotic area ratio. (**D**) TMZ-only, OLE-only, and OLE phenolics-dependent changes in the RNA expression of GSC marker genes CD133 and OCT4 in T98G and A172 cells. *p*-values were calculated using two-way ANOVA for tumorsphere formation and an independent samples t-test for RT-qPCR * *p* < 0.05, ** *p* < 0.0001 compared to untreated cells; n = 3. UNT: untreated, TMZ: temozolomide, OLE: *Olea europaea* leaf extract, OL: oleuropein, HT: hydroxytyrosol, TYR: tyrosol.

The tumorsphere formation assay was used to assess the stem-cell-like characteristics of T98G and A172 cells after treatment with TMZ-only, OLE-only, or OLE phenolics. Similar to our cell proliferation and viability findings, TMZ-only (in T98G: 1.18-fold, *p* < 00001; in A172: 1.38-fold, *p* < 00001) and OLE-only (in T98G: 2.13-fold, *p* < 00001; in A172: 1.5-fold, *p* < 0.0001) decreased tumorsphere size compared to untreated controls (Figure 7A,B). In addition, the necrotic core regions of the OLE-only treated spheroids were smaller than untreated tumorspheres (Figure 7C), which indicates that OLE-only treatment attenuates the development and maintenance of GB stem-like cells (GSCs) via hypoxia [27,28].

Of the OLE phenolics, OL suppressed the sphere size and the necrotic core region the most (in T98G: 4.1-fold, *p* < 00001; in A172: 2.5-fold, *p* < 00001) (Figure 7A–C). Although they were less potent, TYR and rutin demonstrated a similar effect with OL, decreasing the tumorsphere size of T98G cells more effectively compared to A172 cells. In T98G cells, TYR and rutin decreased the tumorsphere sizes 2- and 1.7-fold, respectively, while a 1.3-fold reduction by both phenolics was demonstrated on the A172 tumorspheres (*p* < 0.0001, Figure 7A,B). Likewise, HT was more effective in decreasing the tumorsphere size of T98G cells (1.6-fold) compared to its effect on A172 cells (1.9-fold; *p* < 0.0001, Figure 7A,B). All OLE phenolics reduced the formation of hypoxia-induced necrosis in the tumor core region relative to the untreated tumorspheres. However, rutin’s capacity to interfere with hypoxia was lower relative to the others (Figure 7C).

Likewise, confirming our previous findings in GSCs [29], OLE reduced the RNA expression of CD133 and OCT4 compared to untreated and TMZ-treated T98G and A172 cells (Figure 7D). Among OLE phenolics, OL and HT decreased CD133 and OCT4 RNA expressions at a similar rate to OLE (Figure 7D). In contrast, although TYR and rutin decreased the RNA of CD133 and OCT4 compared to untreated cells, the level of suppression was cell-type dependent (Figure 7D; Table S1).

**Figure 8:**
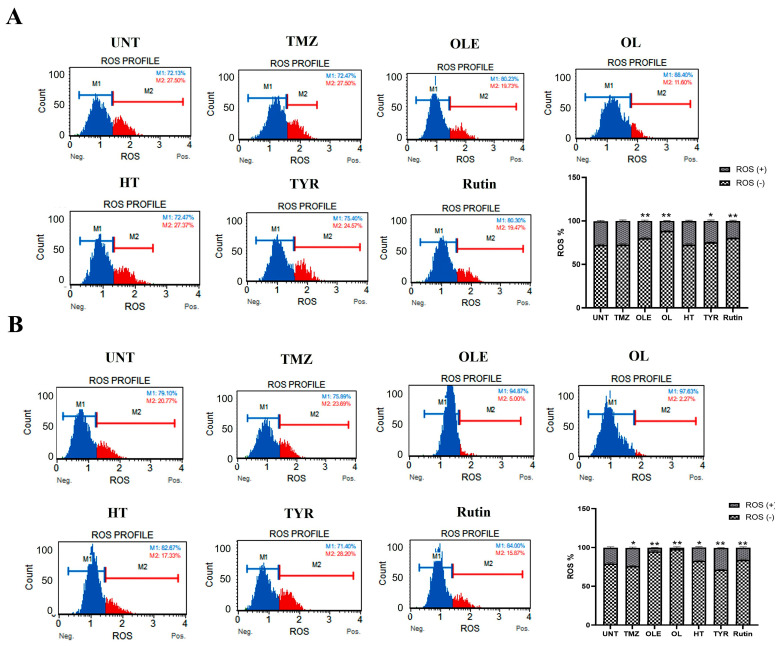
ROS level measured in GB cells. TMZ−only, OLE−only, and OLE phenolic−pretreated T98G (**A**) and A172 (**B**) cells were exposed to dihydroethidium for 30 min. Data are presented as the mean ± SD. *p*-value was calculated as compared to untreated cells using a two-way ANOVA test (n = 3). Statistical significance is shown as * *p* < 0.05, ** *p* < 0.0001. M1: ROS (−), M2: ROS (+). UNT: untreated, TMZ: temozolomide, OLE: *Olea europaea* leaf extract, OL: oleuropein, HT: hydroxytyrosol, TYR: tyrosol.

The ROS increases during the formation of tumor hypoxia [30]. Therefore, we evaluated the effect of OLE and OLE phenolics on the ROS production capacity of GB cells. According to our findings, TMZ treatment alone did not affect the ROS production of T98G cells. In contrast, OLE-only and the OLE phenolics OL and rutin decreased ROS production compared to untreated T98G and A172 cells (Figure 8A,B, *p* < 0.0001). Interestingly, HT reduced ROS production in A172 cells (*p* = 0.0007) but not in T98G cells (Figure 8A,B). In addition, TYR reduced ROS levels in T98G (*p* = 0.002) but did not affect the A172 cells (Figure 8A,B).

### 3.5. OLE Phenolics Slowed down the Cell Migration Rate

**Figure 9:**
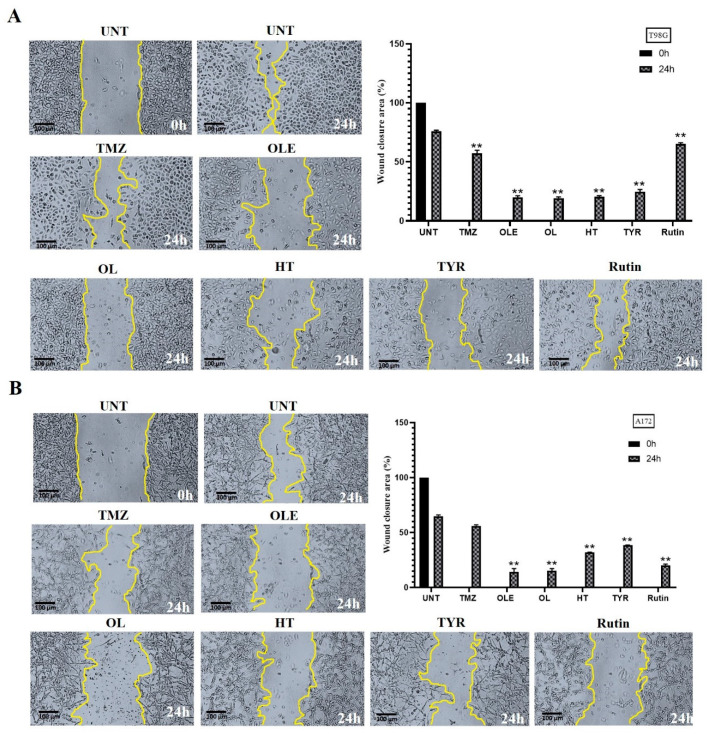
Cell migration was analyzed by wound-healing assay. TMZ−only, OLE−only, and OLE phenolics reduced the cell migration of (**A**) T98G and (**B**) A172 cells compared to the untreated cells. The edges of the wound area are marked in yellow. The size of the wounded area was initially imaged untreated, then 24 h after treatments with TMZ−only, OLE−only, and OLE phenolics in 40x magnification. ImageJ software measured the size of the captured wounded area. *p*-values were calculated using a dependent sample t-test. Data are shown as mean ± SD. ** *p* < 0.0001. UNT: untreated, TMZ: temozolomide, OLE: *Olea europaea* leaf extract, OL: oleuropein, HT: hydroxytyrosol, TYR: tyrosol.

The effect of OLE phenolics on the two-dimensional migration capacity of T98G and A172 cells was evaluated by measuring the wound-healing rate after treatment with OLE phenolics. A total of 75.84% and 65.03% of the wounded area of untreated T98G and A172 cells was closed within 24 h, respectively. TMZ-only slowed the wound-healing rate to 56.96% in T98G cells (Figure 9A) and 55.14% in A172 cells (Figure 9B). Likewise, in T98G and A172 cells treated with OLE-only, 18.14% and 14.25% of the wounded area closed within 24 h (Figure 9A,B). The OLE phenolics, OL, HT, TYR, and rutin healed the scratched area by 20.33%, 21.4%, 22.66%, and 64.25% in T98G cells (Figure 9A) and by 15.22%, 32.00%, 38.5%, and 20.3% in A172 cells (Figure 9B), respectively.

### 3.6. OLE Phenolics Exert an Additive Effect in Combination with TMZ

**Figure 10:**
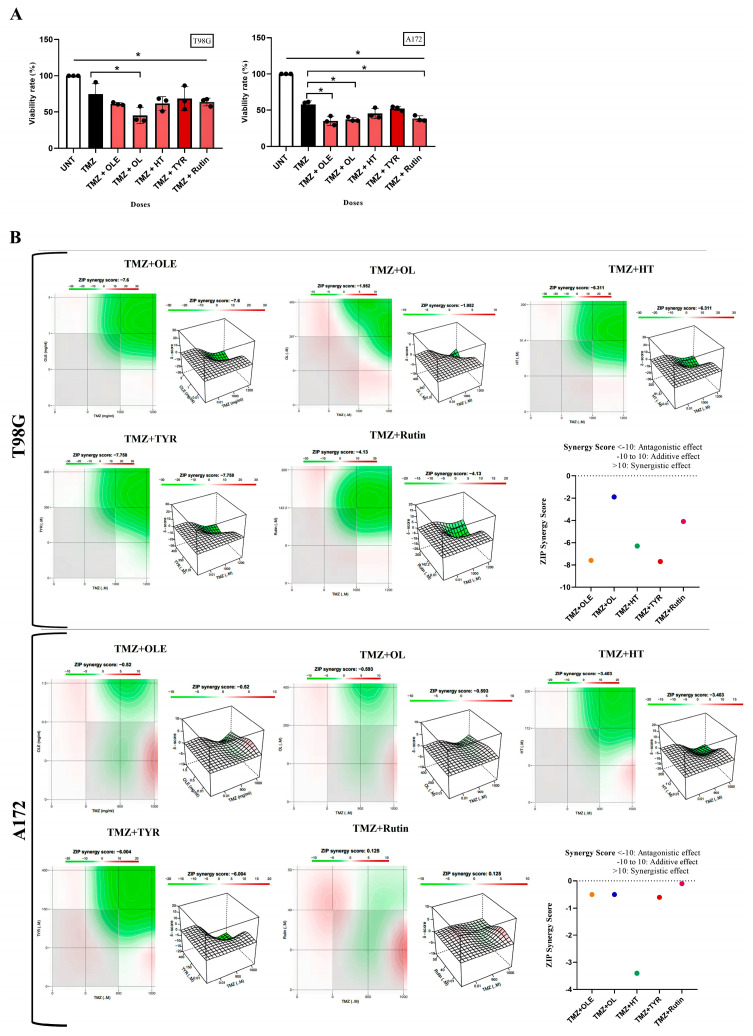
The combined effect of TMZ with OLE and OLE phenolics. (**A**) A WST−1 test detected the effect of TMZ + OLE and TMZ + OLE phenolics on proliferation of T98G and A172 cells. *p*-values were calculated using one-way ANOVA and Tukey′s post hoc tests (n = 3). Data are presented as mean ± SD. *: *p* < 0.05 versus TMZ−only treated and untreated cells. (**B**) The ZIP synergy scores caused by TMZ + OLE, TMZ + OL, TMZ + HT, TMZ + TYR, and TMZ + rutin. UNT: untreated, TMZ: temozolomide, OLE: *Olea europaea* leaf extract, OL: oleuropein, HT: hydroxytyrosol, TYR: tyrosol.

T98G and A172 cells were treated with OLE or OLE phenolics simultaneously with TMZ. While OLE + TMZ treatment decreased the cell proliferation of T98G and A172 cells by 60.56% and 35.2% compared to TMZ-only treatment, TMZ + OL, TMZ + HT, TMZ + TYR, and TMZ + rutin treatments decreased the proliferation of T98G cells by 45.1%, 61.7%, 68.5%, and 63.8% and A172 cells by 37.3%, 45.4%, 52.4%, and 38.2% compared to TMZ-only treatment (Figure 10A), respectively. In addition, the zero-interaction potency (ZIP) synergy score of OLE (T98G_SZIP_ = −7.6; A172_SZIP_ = −0.5), OL (T98G_SZIP_ = −1.9; A172_SZIP_ = −0.5), HT (T98G_SZIP_ = −6.3; A172_SZIP_ = −3.4), TYR (T98G_SZIP_ = −7.7; A172_SZIP_ = −6.0), and rutin (T98G_SZIP_ = −4.1; A172_SZIP_ = −0.1) predicted an additive effect when they were simultaneously used with TMZ (Figure 10B). With this, in T98G cells, the synergy scores between TMZ + OL and TMZ + rutin were higher than TMZ + OLE, whereas these combinations did not cause a difference in the synergy score of TMZ + OLE in A172 cells. In contrast, the synergy scores from TMZ + HT and TMZ + TYR were cell-type dependent. While TMZ + HT resulted in a higher synergy score than TMZ + OLE in T98G cells, its effect was the opposite in A172 cells. Likewise, while the synergy score caused by TMZ + TYR was similar to TMZ + OLE in T98G cells, it was higher than TMZ + OLE in A172 cells (Figure 10B).

**Figure 11:**
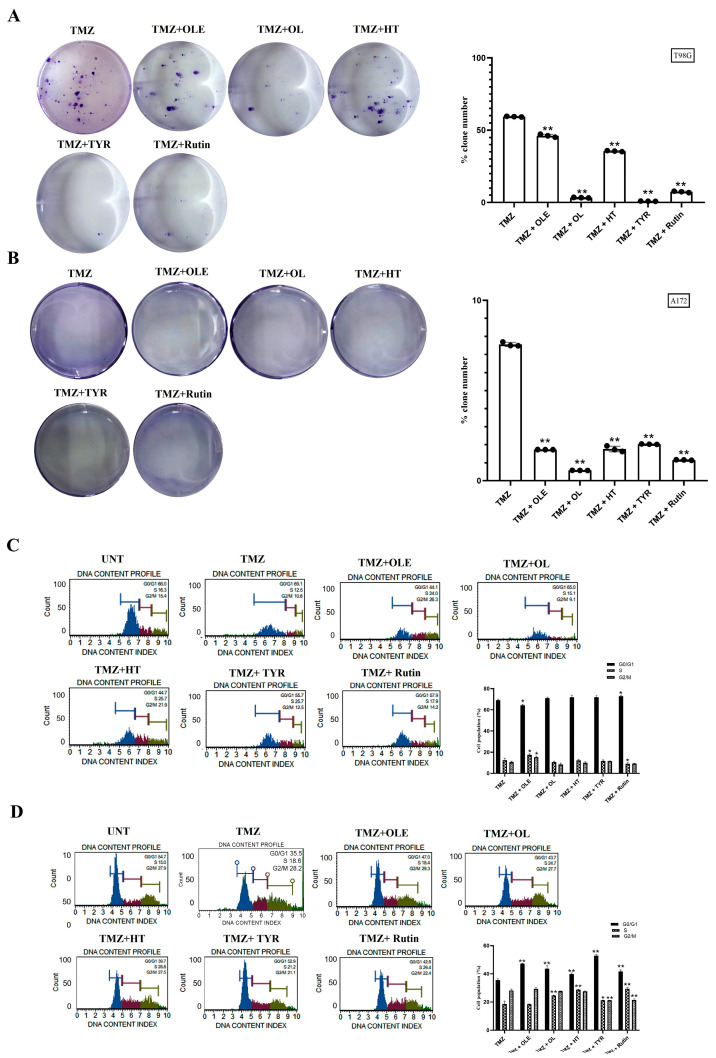
OLE and OLE phenolics additively increased TMZ suppressive capacity on the colony-forming ability of GB cells and promoted its interfering capacity on cell cycle phases. Colony-formation assays were performed using T98G and A172 cells after cotreatment with TMZ and OLE or OLE phenolics. The colony-forming ability of T98G cells (**A**) and A172 cells (**B**) following treatment with TMZ-only, TMZ + OLE, TMZ + OL, TMZ+ HT, TMZ + TYR, and TMZ + rutin. The number of colonies formed by TMZ-only treated GB cells was normalized to 100% to assess the additive effect of the OLE phenolics on TMZ-induced colony reduction. The percentage of the number of formed colonies are graphed. The G0/G1, S, and G2/M phase proportions of T98G and A172 cells (**C**,**D**) (n = 3). Data are presented as the mean ± SD. *p*-value was calculated as compared to untreated cells using the two-way ANOVA test (n = 3). * *p* < 0.05; ** *p* < 0.0001 versus TMZ-treated cells. UNT: untreated, TMZ: temozolomide, OLE: *Olea europaea* leaf extract, OL: oleuropein, HT: hydroxytyrosol, TYR: tyrosol.

The additive effect of OLE phenolics was confirmed by measuring their effect on the colony-forming capacity of GB cells when combined with TMZ. Data showed that TMZ + OL (*p* < 0.0001) and TMZ + HT (*p* < 0.0001) decreased the number of colonies formed by TMZ-only-treated T98G cells (Figure 11A), where colony formation was not observed in A172 cells after treatment with TMZ + OL and TMZ + HT (Figure 11B). In contrast, TMZ + TYR and TMZ + rutin completely suppressed colony formation in both T98G and A172 cells (Figure 11A,B). The effect of OLE in reducing GB cell proliferation, particularly on cell cycle phases, becomes more evident when combined with TMZ (Figure 11C,D). Similar to the single effect of OLE, the combination of TMZ + OLE increased the number of A172 cells in the G0/G1 phase (*p* < 0.0001) compared to those of TMZ-only cells (Figure 11D). However, among the OLE phenolic components, only the combined use of rutin with TMZ reduced the G2/M phase compared to TMZ-only in A172 cells (Figure 11D). Additionally, although TMZ + OL, TMZ + HT, and TMZ + TYR resulted in a decreased number of cells in the S and G2/M phases in T98G cells relative to TMZ + OLE, the distribution of cell cycle phases from TMZ-only treatment was not comparably demonstrated in T98G cells (Figure 11C,D).

### 3.7. TMZ + OL Cell-Type-Independent and TMZ + HT and TMZ + Rutin Cell-Type-Dependent Apoptosis Induction

**Figure 12:**
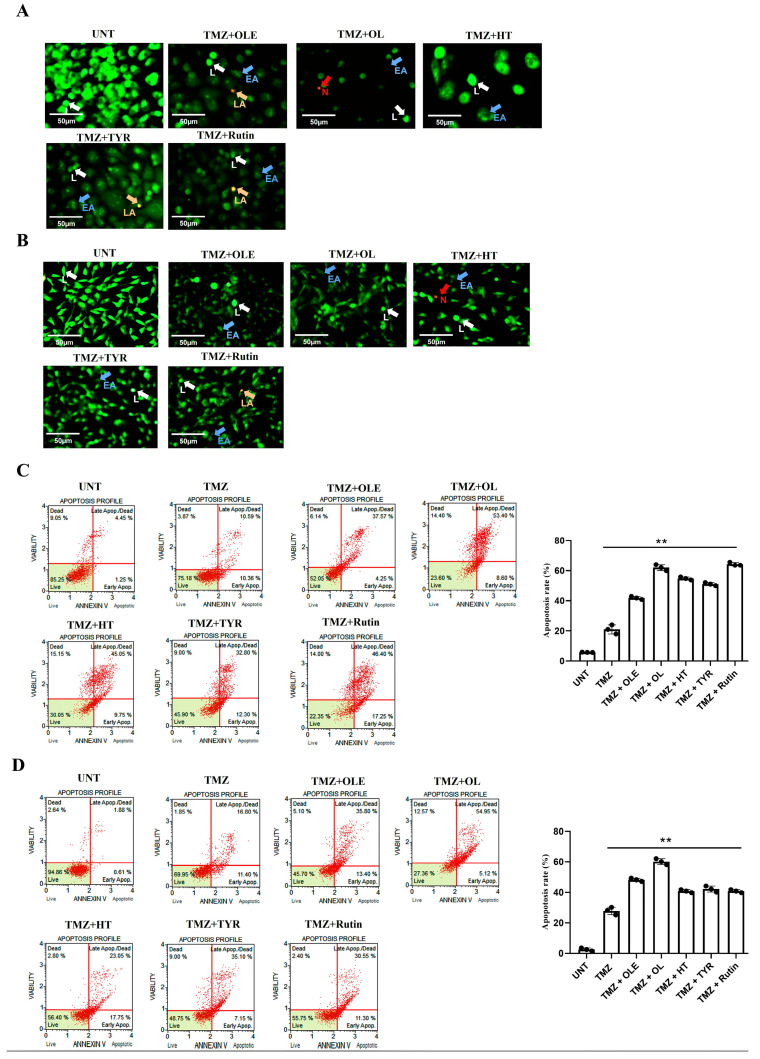
Viability of human GB cells after treatment with TMZ + OLE, TMZ + OL, TMZ + HT, TMZ + TYR, and TMZ + rutin. AO/PI staining of T98G (**A**) and A172 cells (**B**). The green cells with a granular nucleus located on one side indicate apoptosis. In contrast, a circular nucleus uniformly distributed in the center of the cell indicate a cell in interphase. The red cells with an inapparent outline indicate necrosis, dissolved or near disintegration. The color-coded arrows indicate the following: alive cells in white, early apoptosis in blue, late apoptosis in orange, and necrosis in red. (**C**) TMZ-only, TMZ + OLE, and TMZ + OLE phenolics induced apoptosis in T98G and (**D**) A172 cells. The adjusted *p*-values were calculated using one-way ANOVA and Tukey’s post hoc tests. ** *p* < 0.0001 compared to untreated cells; n = 3. UNT: untreated, TMZ: temozolomide, OLE: *Olea europaea* leaf extract, OL: oleuropein, HT: hydroxytyrosol, TYR: tyrosol. L: alive cells, EA: early apoptosis, LA: late apoptosis, N: necrosis.

The AO/PI staining showed that treatment with OLE and its phenolics in combination with TMZ induced apoptotic cell death in T98G and A172 cells (Figure 12A,B). Likewise, TMZ + OLE increased the number of cells undergoing apoptosis compared to TMZ-only in T98G (*p* < 0.0001, Figure 12C) and A172 cells (*p* < 0.0001, Figure 12D). A greater degree of apoptosis was observed with TMZ + OL relative to TMZ (*p* < 0.0001) and was more effective than TMZ + OLE (*p* < 0.0001) in both cell lines (Figure 12C,D). It was noted that the apoptosis-inducing effect of TMZ + HT, TMZ + TYR, and TMZ + rutin was cell-type dependent. TMZ + HT and TMZ + rutin induced apoptosis compared to TMZ-only in both T98G and A172 cells. However, while these were more effective in T98G cells, a lesser apoptosis-inducing effect was demonstrated in A172 cells relative to TMZ + OLE. Conversely, TMZ-only did not affect the viability of T98G and A172 cells compared to TMZ + OLE-treated cells.

### 3.8. Phenolic Compounds Contributed to the Inhibitory Effect of TMZ on GSC Maintenance

**Figure 13:**
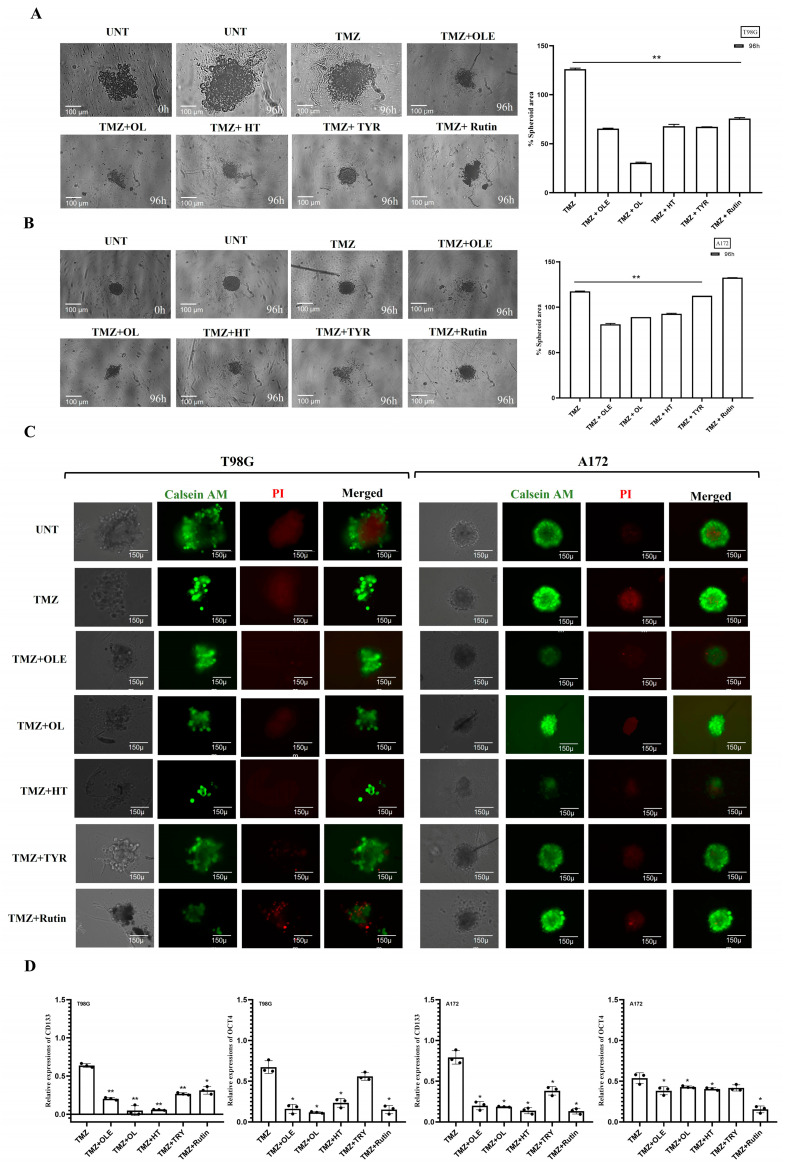
The additive effect of OLE phenolics on GSC inhibitory capacity of TMZ. (**A**) The changes in tumorspheres in T98G cells and (**B**) A172 cells. (**C**) Hypoxia in tumorspheres formed by T98G and A172 cells. The metabolically viable and proliferating cells are shown in green, while the hypoxia-induced necrotic cells are shown in red. The tumorspheres were initially imaged untreated, then 96 h after treatments with TMZ-only, TMZ + OLE, and TMZ + OLE phenolics at 40× magnification. ImageJ software measured the size of the captured tumorspheres and the viable/necrotic area ratio. (**D**) Changes in the RNA expression of GSC marker genes in GB cells. *p*-values were calculated using an independent samples *t*-test. * *p* < 0.05, ** *p* < 0.0001 compared to untreated cells; n = 3. UNT: untreated, TMZ: temozolomide, OLE: *Olea europaea* leaf extract, OL: oleuropein, HT: hydroxytyrosol, TYR: tyrosol.

Supporting our previous findings [16], the combined use of TMZ and OLE reduced the size of tumorspheres and the hypoxia at the core site of the tumorsphere formed by T98G and A172 cells (Figure 13A–C).

OLE phenolics’ individual T98G and A172 cell line tumorsphere suppression capacity was reflected in their combined effect with TMZ. TMZ + OL led to a significant reduction in tumorsphere growth (Figure 13A,B) and attenuated the hypoxic core site compared to TMZ-treated tumorspheres (Figure 13C). In addition, TMZ + HT and TMZ + TYR decreased the size of tumorspheres and diminished the sphere integrity compared to TMZ-only treated tumorspheres (*p* < 0.0001, Figure 13A,B). The hypoxic core site was likewise reduced in TMZ + HT- and TMZ + TYR-treated tumorspheres (Figure 13C). The effect of TMZ + rutin on the inhibition of the size of tumorsphere was similar to the effect of TMZ + HT and TMZ + TYR in T98G cells (Figure 13A). In contrast, while TMZ + rutin did not affect the tumorsphere size in A172 cells, it decreased the tumorsphere cell density (Figure 13B). TMZ + rutin also reduced hypoxia, albeit to a lesser extent compared to the combined effect of other phenolics with TMZ (Figure 13C).

Cotreatment with TMZ + OL and TMZ + HT demonstrated an enhanced inhibitory capacity on CD133 and OCT4 RNA expressions in T98G and A172 cells compared to TMZ alone (Figure 13D). Interestingly, while rutin-only failed to outperform OLE to suppress CD133 RNA expression in T98G and A172 cells, the combined usage of TMZ + rutin resulted in a significant attenuation in the CD133 RNA level of A172 cells (*p* < 0.05; Figure 13D). Moreover, TMZ + rutin considerably suppressed the expression of OCT4 RNA in both cell lines. Conversely, while TMZ + TYR improved the CD133 and OCT4 inhibitory capacity, the effect of TYR was less than the other phenolics.

**Figure 14:**
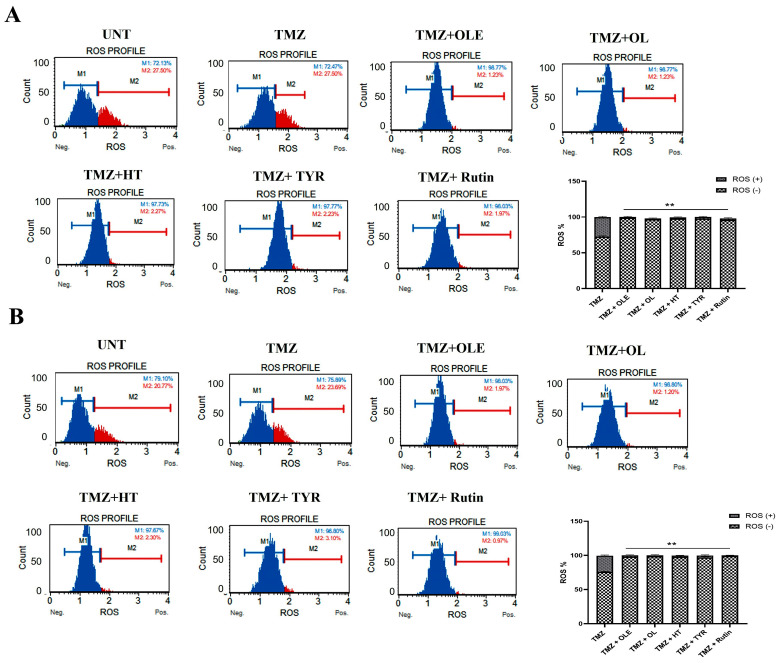
ROS levels in (**A**) T98G and (**B**) A172 cells treated with TMZ−only, TMZ + OLE, and TMZ + OLE phenolics. Data are presented as the mean ± SD. *p*-value was calculated as compared to TMZ-only treated cells using a two-way ANOVA test (n = 3). ** *p* < 0.0001. M1: ROS (−), M2: ROS (+). UNT: untreated, TMZ: temozolomide, OLE: *Olea europaea* leaf extract, OL: oleuropein, HT: hydroxytyrosol, TYR: tyrosol.

Examining the level of hypoxia induced by the combined treatment of TMZ + OLE and TMZ + OLE phenolics, the amount of ROS was significantly reduced after the coadministration of OLE or each OLE phenolic component with TMZ relative to the ROS production caused by TMZ-only in both T98G and A172 cells (Figure 14A,B).

### 3.9. OLE Phenolics Induced the Inhibitory Effect of TMZ on GB Cell Migration

**Figure 15:**
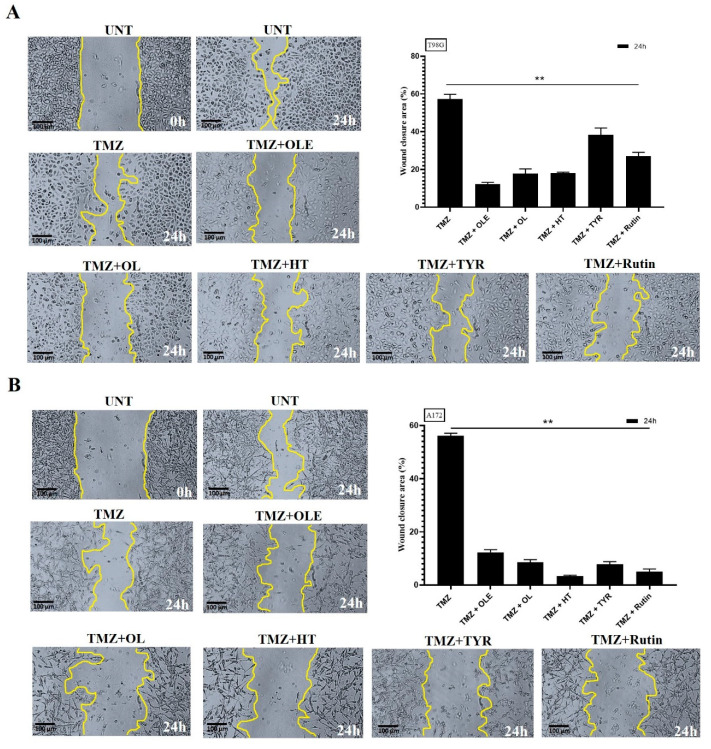
The additive effect of OLE phenolics on GB cell migration capacity of TMZ. TMZ-only, TMZ + OLE, and TMZ + OLE phenolics reduced the cell migration of (**A**) T98G and (**B**) A172 cells compared to the TMZ-only treated cells. The edges of the wound area are marked in yellow. The size of the wounded area was initially imaged untreated, then 24 h after treatments with TMZ-only, TMZ + OLE, and TMZ + OLE phenolics at 40× magnification. ImageJ software measured the size of the captured wounded area. *p*-values were calculated using a dependent sample T-test. Data are shown as mean ± SD. ** *p* < 0.0001. UNT: untreated, TMZ: temozolomide, OLE: *Olea europaea* leaf extract, OL: oleuropein, HT: hydroxytyrosol, TYR: tyrosol.

Upon a combined treatment with TMZ and OLE phenolics, the two-dimensional migration capacity of TMZ-treated GB cells was reduced by 56.96% and 55.14% of the wounded area of TMZ-only treated T98G and A172 cells, respectively, and were closed within 24 h (Figure 15A,B). TMZ + OLE slowed the wound-healing rate to 13.18% in T98G (Figure 15A) and 12.13% in A172 cells (Figure 15B). In addition, upon combined treatment with TMZ and OLE phenolics, 20.08%, 18.31%, 35.38%, and 25.1% of the wounded area in T98G cells (Figure 15A), and 8.55%, 3.3%, 7.8%, and 6.03% in A172 cells (Figure 15B), were recovered by TMZ + OL, TMZ + HT, TMZ + TYR, and TMZ + rutin, respectively.

## 4. Discussion

T98G and A172 cells are heterogeneous since their phenotypic features also impact their growth characteristics [31]. While both T98G and A172 cells are fibroblast-like, the former is polymorphic with polygonal and spherical cells, while the latter is monomorphic [31]. MGMT is expressed in both cell lines. However, this is much higher in T98G cells such that TMZ resistance is demonstrated, while only a low basal amount is observed in A172 [32,33]. In addition, in contrast to A172 cells, T98G cells express the S100 protein that functions as an intracellular Ca^2+^ sensor, which contributes to tumorigenic processes and facilitates drug resistance [34]. However, A172 cells exhibit a higher level of the GSC marker CD133/2, mesenchymal markers CD90 and CD105, fibroblast activation protein, and tenascin C, which exert protumorigenic functions in the formation of tumor stroma [31,35]. Although both cell lines express VEGF and FGF2, the expression of these proteins is higher in A172 [31]. It is important to note, however, that the GSC phenotype via CD133 expression was found to be unrelated to TMZ resistance in GB cell lines, suggesting that GSC content and angiogenic features are prominent in A172 cells, while T98G cells are highly differentiated and exhibit resistance to TMZ [31,33].

Our previous findings suggest that OLE increases the cancer cell-killing effect of TMZ against GB and GSCs by modulating microRNA (miRNA) expression [18,29]. In addition, it was previously demonstrated that OLE improves the TMZ response of GB tumors by inducing MGMT methylation [36]. The current study supports our previous findings where the combined treatment of OLE with TMZ decreased the proliferation of both T98G and A172 cells relative to TMZ-only treatment, regardless of their difference in TMZ sensitivity capacity.

OL is recognized as the most abundant phenolic of OLE [37]. The anticancer effect of OL as an antiproliferative and an apoptosis promoter was previously demonstrated in multiple cancer types, such as breast and colon cancer cell lines [38,39,40,41]. Additionally, our previous study showed that an equal concentration of OL-only from a predetermined amount of OLE (15%) demonstrated an anticancer effect in T98G cells [17]. For the current study, however, we optimized the IC50 concentration of OL for T98G and A172 cells. As expected, OL-only reduced the cell proliferation capacity and colony growth and induced apoptosis in both T98G and A172 cells independent from tumor features. In addition, this effect of OL-only was more evident compared to the IC50 concentration of OLE-only. Our previous study elucidated that OL induces Let-7d expression in T98G cells [17]. Let-7 is recognized as one of the regulatory miRNA families of differentiation, pluripotency, and apoptosis [42]. In support of this, OL-only decreased the expression of GSC marker genes, including *CD133* and *OCT4*, in both cell lines. These findings suggest that the OL effect alone could play a substantial role in the action of OLE against GB cells and has the potential to be used in GB therapy research independent of other phenolics in the OLE structure. In line with this, the additive effect on TMZ by the IC50 concentration of OL in inhibiting GB cell proliferation and colony formation was more potent than OLE, and the apoptosis-promoting effect of TMZ + OL was the highest among all OLE phenolics. Given that the decrease in the expression of GSC markers was remarkable in T98G cells after TMZ + OL treatment and that it was as effective as TMZ + OLE in A172 cells, it can be surmised that the GSC inhibitory effect of the combined use of TMZ + OLE could be due to the GSC inhibitory effect of OL within the content of OLE.

HT is the second major OLE structure phenol [37], where the degradation of OL leads to the majority of its production [43]. The antiproliferative, proapoptotic, and anti-inflammatory properties of HT were recently evidenced in human neuroblastoma cells (SH-SY5Y), acute human leukemia T-cells (Jurkat and HL60), and colorectal cancer cells (HCT116 and LoVo) [44,45,46]. In this study, HT led to 50% inhibition of GB cell proliferation in the lowest concentration among all investigated phenolics of OLE by arresting the cell cycle in the S and G2/M phases, demonstrating its robust preventive capacity against GB. In addition, HT was similar to OL in anti-GB cell properties. However, OL alone showed as much or even more efficacy compared to OLE against GB, while the effect of HT was weaker than the overall effect of OLE. In addition, HT was more effective in promoting apoptosis in T98G cells than in A172 cells, evidenced by HT effectively reducing GSC biomarkers, CD133 and OCT4, in T98G cells. In contrast, it could attenuate the expression of these marker genes at a lower level in A172 cells, which express these markers more prominently [31]. Although HT was less effective than OL in reducing the GSC phenotype, HT could effectively decrease the size of tumorspheres derived from A172 cells. Tumorsphere models mimic tumor tissues that mutually consist of nonproliferating but viable hypoxic tumor cells in the central region and have CSC expansion [47]. The cytosolic ROS plays a role in stabilizing hypoxia-inducible factor signaling and maintenances hypoxic conditions where the GSC population can reside [48]. In this study, HT reduced ROS production and decreased the hypoxic region of the tumorspheres from both T98G and A172 cells. Studies showed that a loss in the hypoxia-inducible factors attenuates the maintenance of GSCs [48,49]. Our findings suggest that HT could target GSC cells by attenuating cellular ROS levels. Thus, HT could be one of the responsible phenolics of the GSC-targeting effect of OLE. Indeed, when HT was combined with TMZ treatment, it contributed to the GSC suppressor capacity of TMZ, which led to one of the most significant reductions in CD133 stem cell marker expression, level of ROS production, and size of the hypoxic core site in tumorspheres of A172 cells among the investigated OLE phenolics. However, the apoptosis promoter effect of TMZ + HT was above the impact of TMZ + OLE in T98G cells, which are more resistant to TMZ. This suggests that unlike OL and despite being one of the most prominent anti-GB phenolics of OLE, it could not represent the full impact of OLE against GB.

In addition to HT, TYR is one of the degradation products of OL [40]. Its antioxidant capacity has been demonstrated by several studies [50,51,52]. While HT demonstrated an anti-GB effect similar to OL in this study, TYR was the least effective. Its effect on colony-forming capacity and arresting cell cycle was the least among all of the investigated OLE phenolics.

A study by Goldsmith and colleagues reported the lack of influence of TYR on the viability of pancreatic cancer cells, including MIA PaCa-2, BxPC-3, and CFPAC-1 [53]. Although TYR promoted apoptosis, this was less than the effect of OLE and was the weakest among all investigated OLE phenolics. In addition, TYR resulted in a poor reduction in the size of the hypoxic core site of tumorspheres and did not affect the degree of ROS production. Furthermore, its migration inhibitory capacity was limited compared to OLE, OL, and its degradation product HT. Therefore, current findings suggest that the GB inhibitory effect of OLE is more likely to originate from other OLE phenolics than TYR.

Nevertheless, in contrast to the single effect of TYR, its unexpected additive effect on TMZ therapy should not be ignored. In our study, the combined use of TMZ + TYR contributed to the anticancer effect of TMZ in terms of reducing ROS production and controlling cancer cell spread by inhibiting the migration of GB cells. The current analysis could not explain the effect of TYR on TMZ against ROS and GB migration, warranting further validation analyses to clarify this mechanism. On the other hand, based on our findings, the GB cell proliferation reduction and apoptosis induction capacity of TMZ + TYR were the least among the TMZ combinations with other OLE phenolics. TYR exhibited an antigenotoxic effect against spontaneous DNA damage [54,55]. Considering that the mechanism of action of TMZ is that it triggers apoptosis in cells by damaging DNA, the inhibitory effect of TYR on DNA damage may lead to a relatively smaller impact on cell proliferation and viability compared to other TMZ + OLE phenolic combinations [56].

Rutin was the fourth investigated antioxidant phenolic of OLE. Its anticancer effect is attributed to the inhibition of lipid peroxidation and reduction of oxidative stress [57,58]. Lipid peroxidation is induced by ROS damage of high polyunsaturated fatty acids in cellular membranes [59] and was shown to prime apoptotic signals by increasing NF-κB activity while decreasing IκB degradation and inducing the phosphorylation of antiapoptotic Bcl-2 [60,61]. In support of this, rutin decreased the ROS production in both T98G and A172 cell lines in the current study, but its apoptosis-inducing effect was lower than OL and HT. Sgarbi and colleagues showed that hypoxic conditions stimulate ROS production in cells not expressing the endogenous inhibitor protein 1 (IF1) of the ATP synthase complex. However, in the presence of IF1 expression, hypoxia reduces ROS production to regulate the energy metabolism of cancer cells [62]. We found that rutin was insufficient in reducing hypoxia and GSC phenotype level compared to other OLE phenolics. Therefore, the presence of hypoxia may also promote the reduction of ROS levels in rutin-treated GB cells.

Nevertheless, rutin inhibited the formation of colonies and reduced the swiftness of cell migration in a monolayer cell culture for 24 h. In addition, it slowed down the cell cycle in the S and G2/M phases. Studies showed that rutin arrests the signaling pathways that promote cancer cell proliferation, such as mitogen-activated protein kinase, PI3K/Akt, Wnt/β-catenin, and epidermal growth factor signaling pathways by suppressing the transcription of their activators [57,63,64,65]. While rutin could inhibit colony formation by arresting GB cell proliferation because it is insufficient to reduce GSC phenotype, its long-term effect on tumor relapse remains unclear with the current data.

In previous studies, the combination of rutin with chemotherapy drugs, including tamoxifen, cisplatin, 5-Fluorouracil, oxaliplatin, and doxorubicin, was suggested to reduce drug resistance and chemotherapy side effects [58,66,67,68,69]. In our study, rutin displayed an additive effect on TMZ therapy, suggesting that it does not augment the mechanism of action of chemotherapy drugs but independently induces ROS and arrests the cell cycle. This is evidenced by an enhanced anticancer response in combined treatment with rutin and chemotherapy drugs. Supporting this hypothesis, the treatment with TMZ + rutin led to a more pronounced degree of ROS inhibition and increased the degree of migration capacity loss in GB cells compared to TMZ-only. In addition, it interfered with the cell cycle in the G2/M phases and slowed down the proliferation of GB cells in a more determined way. However, because rutin could not affect GSC phenotype, it could not contribute to the effect of TMZ on GSC in A172 cells, which strongly express GSC markers. Although rutin itself is not as effective as OLE, a combined treatment with TMZ and rutin can potentially increase treatment success in GB.

## 5. Conclusions

This study first investigated the effect of OLE phenolics on GB cells and their additive potential to TMZ therapy. OL, the most abundant phenolic in OLE, is as effective as OLE in GB cells. Because OL demonstrates additive effects that can augment the effect of TMZ, further research is warranted to clarify its potential to be an individual anticancer drug against GB. HT, the second abundant phenolic found in the OLE structure, is less effective in impacting GB cells compared to OL. However, considering its potential to decrease the GSC phenotype, it could be suggested as a strong candidate phenolic to be used as an additive to TMZ treatment. The effect of rutin alone on GB cells is insufficient to generate a treatment potential. However, its utility in combination with TMZ can be evaluated in further studies. Lastly, although the simultaneous use of TYR with TMZ slightly increased the success of TMZ treatment, this effect was not as strong as other phenolics. While TYR alone was not effective, in combination with TMZ, it decreased the ROS production in GB cells via a mechanism that could not be fully explained in this study. Considering its weak effect against GB, it is not suggested as among the priority phenolics of OLE that can be used to develop GB treatment.

## Data Availability

All data generated or analyzed during this study are included in this published article. The data that support the findings ofthis studyareavailable from thecorresponding author upon request.

## Supplementary Materials

The following supporting information can be downloaded at: https://www.mdpi.com/article/10.3390/life13020470/s1. Figure S1. Only OLE and OLE phenolics affect L929 cell proliferation, depending on the active dose. UNT: Untreated, OLE: Olea europaea leaf extract, OL: Oleuropein, HT: Hydroxytyrosol, TYR: Tyrosol. Table S1. Expression levels of stem-like cancer cell-related marker genes in T98G and A172 cells
