# Supplementary material for: Olea europaea Leaf Phenolics Oleuropein, Hydroxytyrosol, Tyrosol, and Rutin Induce Apoptosis and Additionally Affect Temozolomide against Glioblastoma: In Particular, Oleuropein Inhibits Spheroid Growth by Attenuating Stem-like Cell Phenotype
Source: Life (Basel). 2023 Feb 8;13(2):470. doi: 10.3390/life13020470 (PMC9964321; doi:10.3390/life13020470)
Supplement: Supplementary file 1 [file life-13-00470-s001.zip › Table S1.pdf]

Table S1. Expression levels of stem-like cancer cell-related marker genes in T98G and A172 cells

	T98G cells		A172 cells	
**Doses**	***CD133***	***OCT4***	***CD133***	***OCT4***
TMZ				
2^-ΔCT	0.61622	0.63299	0.88533	0.46184
Fold change	-1.56	-1.48	-1.26	-1.85
p value	*p<0.0001*	*p=0.0019*	*p=0.0123*	*p=0.0003*
OLE				
2^-ΔCT	0.06448	0.23707	0.42190	0.57474
Fold change	-16.26	-5.07	-2.59	-1.78
p value	*p<0.0001*	*p<0.0001*	*p<0.0001*	*p<0.0001*
OL				
2^-ΔCT	0.00697	0.12457	0.35893	0.38915
Fold change	-18.44	-8.25	-3.07	-2.42
p value	*p<0.0001*	*p<0.0001*	*p<0.0001*	*p<0.0001*
HT				
2^-ΔCT	0.02751	0.16330	0.22429	0.62065
Fold change	-17.08	-6.25	-5.31	-1.67
p value	*p<0.0001*	*p<0.0001*	*p<0.0001*	*p<0.0001*
TRY				
2^-ΔCT	0.16677	0.15020	0.38489	0.65178
Fold change	-4.75	-6.11	-2.59	-1.43
p value	*p<0.0001*	*p<0.0001*	*p<0.0001*	*p=0.0005*
Rutin				
2^-ΔCT	0.20902	0.06077	0.40626	0.30958
Fold change	-4.93	-19.15	-3.00	-3.22
p value	*p<0.0001*	*p<0.0001*	*p<0.0001*	*p<0.0001*
TMZ+OLE				
2^-ΔCT	0.21862	0.19879	0.24358	0.41641
Fold change	-4.9	-6.19	-4.99	-2.61
p value	*p<0.0001*	*p<0.0001*	*p<0.0001*	*p<0.0001*
TMZ+OL				
2^-ΔCT	0.00506	0.12724	0.18176	0.42337
Fold change	-20.33	-8.53	-5.35	-2.33
p value	*p<0.0001*	*p<0.0001*	*p<0.0001*	*p<0.0001*
TMZ+HT				
2^-ΔCT	0.06308	0.27292	0.16964	0.40848
Fold change	-16.92	-4.25	-7.22	-2.46
p value	*p<0.0001*	*p<0.0001*	*p<0.0001*	*p<0.0001*
TMZ+TRY				
2^-ΔCT	0.28426	0.54182	0.42272	0.40118
Fold change	-3.73	-1.79	-2.61	-2.39
p value	*p<0.0001*	*p<0.0001*	*p<0.0001*	*p<0.0001*
TMZ+Rutin				
2^-ΔCT	0.36409	0.16485	0.14163	0.16774
Fold change	-3.18	-6.57	-7.54	-6.47
p value	*p<0.0001*	*p<0.0001*	*p<0.0001*	*p<0.0001*
