# Supplementary figures and images for: Olea europaea Leaf Phenolics Oleuropein, Hydroxytyrosol, Tyrosol, and Rutin Induce Apoptosis and Additionally Affect Temozolomide against Glioblastoma: In Particular, Oleuropein Inhibits Spheroid Growth by Attenuating Stem-like Cell Phenotype
Source: Life (Basel). 2023 Feb 8;13(2):470. doi: 10.3390/life13020470 (PMC9964321; doi:10.3390/life13020470)

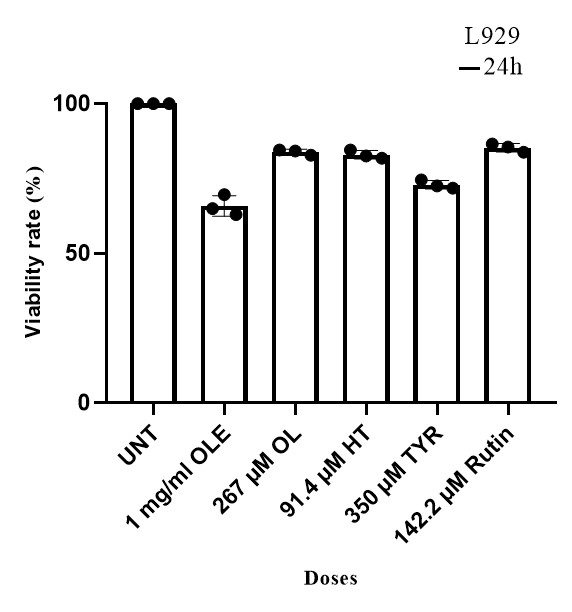

Supplement: Supplementary file 1 [file life-13-00470-s001.zip › Figure S1.jpg]
